# A population-based study of ambulatory and surgical services provided by orthopaedic surgeons for musculoskeletal conditions

**DOI:** 10.1186/1472-6963-9-56

**Published:** 2009-03-31

**Authors:** Mayilee Canizares, Crystal MacKay, Aileen M Davis, Nizar Mahomed, Elizabeth M Badley

**Affiliations:** 1 Arthritis Community Research and Evaluation Unit, Division of Health Care and Outcomes Research, Toronto Western Research Institute, Toronto, Ontario, Canada; 2 Department of Physical Therapy, University of Toronto, Toronto, Ontario, Canada; 3 Department of Physical Therapy, Rehabilitation Science and Health Policy, Management and Evaluation, University of Toronto, Toronto, Ontario, Canada; 4 Adjunct Scientist, Institute for Clinical Evaluative Sciences, Toronto, Ontario, Canada; 5 Department of Surgery, University of Toronto, Toronto, Ontario, Canada; 6 Dalla Lana School of Public Health, University of Toronto, Toronto, Ontario, Canada

## Abstract

**Background:**

The ongoing process of population aging is associated with an increase in prevalence of musculoskeletal conditions with a concomitant increase in the demand of orthopaedic services. Shortages of orthopaedic services have been documented in Canada and elsewhere. This population-based study describes the number of patients seen by orthopaedic surgeons in office and hospital settings to set the scene for the development of strategies that could maximize the availability of orthopaedic resources.

**Methods:**

Administrative data from the Ontario Health Insurance Plan and Canadian Institute for Health Information hospital separation databases for the 2005/06 fiscal year were used to identify individuals accessing orthopaedic services in Ontario, Canada. The number of patients with encounters with orthopaedic surgeons, the number of encounters and the number of surgeries carried out by orthopaedic surgeons were estimated according to condition groups, service location, patient's age and sex.

**Results:**

In 2005/06, over 520,000 Ontarians (41 per 1,000 population) had over 1.3 million encounters with orthopaedic surgeons. Of those 86% were ambulatory encounters and 14% were in hospital encounters. The majority of ambulatory encounters were for an injury or related condition (44%) followed by arthritis and related conditions (37%). Osteoarthritis accounted for 16% of all ambulatory encounters. Orthopaedic surgeons carried out over 140,000 surgeries in 2005/06: joint replacement accounted for 25% of all orthopaedic surgeries, whereas closed repair accounted for 16% and reductions accounted for 21%. Half of the orthopaedic surgeries were for arthritis and related conditions.

**Conclusion:**

The large volume of ambulatory care points to the significant contribution of orthopaedic surgeons to the medical management of chronic musculoskeletal conditions including arthritis and injuries. The findings highlight that surgery is only one component of the work of orthopaedic surgeons in the management of these conditions. Policy makers and orthopaedic surgeons need to be creative in developing strategies to accommodate the growing workload of orthopaedic surgeons without sacrificing quality of care of patients with musculoskeletal conditions.

## Background

Musculoskeletal conditions are the most prevalent chronic conditions in developed countries[1-3] affecting 20% to 40% of the adult population. As the symptoms of the majority of these conditions last more than a year they offer challenges for the right level of provision of care at the right time. These are a diverse group of conditions that include arthritis, soft-tissue disorders (such as bursitis and tendonitis), back pain, and injury to the bones, joints and supporting tissues (such as fractures, dislocations and sprains and strains). This group of conditions has a major financial impact on society. A study of the economic burden of illness to the Canadian society has shown that musculoskeletal conditions were among the most costly chronic conditions with a large proportion of the cost associated with disability and lost productivity [4].

The prevalence of many of these conditions is increasing with the aging of the baby boomer population. For example, the prevalence of arthritis in Canada is estimated to increase from 17% to 26% by 2021 [5], with similar increases projected for the US [6]. Besides aging, increasing rates of obesity in the population are also likely to contribute to an increase in the incidence of osteoarthritis, especially of the knee [7,8]. The number of hip fractures in the population 65 years or older is also expected to increase over the next decades [9,10]. This increase in the number of individuals with musculoskeletal problems will affect the demand for health services in the near future.

Musculoskeletal conditions are managed by many health care professionals ranging from primary to specialist care. Among specialists, orthopaedic surgeons are essential in providing musculoskeletal care. In Canada, 15% of individuals visiting physicians for musculoskeletal conditions saw an orthopaedic surgeon at least once [11] while in the US over 19% of all ambulatory visits for musculoskeletal conditions were made to an orthopaedic surgeon [12]. Nevertheless, despite the major contribution to ambulatory care, much of the attention on the role of orthopaedic surgeons tends to be focused on surgical services, with waiting times and access to operating rooms and associated hospital care being an issue in many jurisdictions [13-15].

Studies of orthopaedic manpower have shown an overall decline in the number of orthopaedic surgeons practicing in Ontario with an increase in their average age [16,17]. The situation in Ontario mirrors the overall picture in Canada where a shortfall of 400 orthopaedic surgeons to meet demand for these services has been reported [13]. Studies in the US have focused on predicting the impact of the aging population on the surgical workforce. Findings indicate that the demand for orthopaedic surgery will increase substantially in the near future and that the US will be facing a shortage of orthopaedic surgeons as it attempts to meet that demand [9,18,19].

These shortages of orthopaedic surgeons represent a major challenge for the healthcare system to provide both timely access and quality care to people with musculoskeletal problems. Therefore, the examination of the clinical practice patterns of orthopaedic surgeons in office and hospital settings is crucial to develop evidence-based strategies in order to maximize the availability of orthopaedic surgical resources within the context of the healthcare services needed by people with musculoskeletal conditions. The purpose of this study was to examine the number and characteristics of patients seen by orthopaedic surgeons in a defined population within one year in ambulatory as well as hospital settings.

## Methods

This is a cross-sectional population-based study of the residents of Ontario, Canada who accessed orthopaedic services in the fiscal year 2005/06 (April 1^st ^through March 31^st^). Ontario has a publicly funded healthcare system which covers all medically necessary physician visits and procedures. Therefore the analyses, which are based on all encounters by Ontarians of all ages with an orthopaedic surgeon in the fiscal year 2005/06, can be considered to be representative of the total population accessing orthopaedic services.

Each encounter represents a discharge of a hospital inpatient, an ambulatory visit to an orthopaedic surgeon, a visit to an emergency department or a day surgery. Encounters in ambulatory settings (e.g. doctor's office, hospital outpatient departments) were identified through the Ontario Health Insurance Plan (OHIP) physician claim database [20]. Encounters that occurred in hospital settings (inpatient, emergency departments or encounters for day surgery) were identified through the Canadian Institute for Health Information (CIHI) hospital separation databases [21].

### Ambulatory encounters

In Ontario, physicians are reimbursed after submitting claims to OHIP for each service provided. The database includes associated diagnosis and individual demographic information. The OHIP diagnostic codes are a subset of codes from the International Classification of Diseases, 9^th ^Revision (ICD-9) [22]. In addition to a diagnostic code, each physician claim also provides a unique patient identification number, doctor identification number, and fee codes based on the type of services/procedures received during the visit. Physician specialty was identified by linking the physician identifier in the claims database to the Ontario Physician Workforce Database, which records all registered physicians in Ontario. Orthopaedic surgeons service claims submitted during the fiscal year 2005/06 were extracted. Ambulatory encounters were obtained by removing claims for associated diagnostic and therapeutic tests and procedures, as well as claims generated during hospitalizations and emergency department visits to avoid duplication when counting the number of encounters. All claims made by the same physician on the same date for the same patient were considered one encounter.

### Hospital encounters

All hospitals in Ontario are required to submit demographic and clinical information about all hospital admissions and discharges, including transfers and deaths, to CIHI. Trained hospital medical records staff transcribe information from each patient's medical chart using standard diagnosis and procedure codes (International Classification of Diseases, 10^th ^revision and Canadian Classification of Health Interventions) [21]. The databases also include patient's age, sex and location of residence as well as hospital-based services (health care providers, procedures and diagnoses), in-hospital outcomes and length of hospital stay. All hospital discharges from individuals from Ontario during the fiscal year 2005/06 that had an orthopaedic surgeon listed in any field corresponding to a health care provider were included for analysis. Hospital encounters included hospital discharges, day surgeries, and visits to emergency departments.

### Condition groups

In both ambulatory and hospital encounters databases equivalent condition groups were created. These groups are based on those used in previous work [5] and developed in consultation with an orthopaedic surgeon (NM). Diagnostic codes were collapsed into four condition groups: arthritis and related conditions, injury and related conditions, bone and joint conditions, and other conditions (Additional file 1). The *arthritis and related *group included conditions like osteoarthritis, rheumatoid arthritis, connective tissue disorders and soft tissue disorders. *Injury and related conditions *included fractures, dislocations, strains, sprains, and other injuries. *Bone and joint conditions *included other conditions of the spine, bone conditions such as foot deformities, and unspecified bone and joint conditions, which are ill-defined symptoms such as leg pain or joint pain. Other conditions include a range of diagnoses not intrinsically related to the musculoskeletal system such as cancer, cellulitis and abscess, and congenital anomalies. In the ambulatory visits database, physicians are allowed to record one diagnostic code per visit. However, in the hospital databases multiple diagnoses can be recorded. Condition groups were assigned based on the most responsible diagnosis (diagnosis that significantly contributed to the length of stay) [21].

### Surgical groups

Data on the type of orthopaedic surgical procedure were obtained from the CIHI databases. Since 2002 surgical procedures are coded using the Canadian Classification of Health Interventions (CCI). This is a classification system that is organized first by body region and then by main types of interventions. As there were no published data on surgical groupings for classification, a comprehensive list of procedures relevant to the musculoskeletal system was created and procedure codes were categorized according to clinical relevance and frequency of the procedures. The groups were created in consultation with an orthopaedic surgeon (NM). Surgical groups were created for six major types of orthopaedic surgery: joint replacement, closed repair (including arthroscopic meniscectomy, arthroscopic debridement), open repair (including repair of ligaments, osteotomy, tendinoplasty), reduction with or without fixation, spinal surgery (fusion, sequestrectomy spinal vertebrae), and other surgery (including amputation, surgical immobilization, and fusion other than spinal).

In addition, the anatomic location of the surgery was recorded and was grouped as follows: hip (pelvis, femoral neck and head, proximal femur), knee (distal femur, knee joint, proximal tibia, patella, and proximal fibula), shoulder and elbow (neck, scapula, clavical, humerus, ulnar head and proximal radius), hand and wrist (distal radius and ulna, carpals bone, metacarpal bones, phalanges), ankle and foot (distal tibia and fibula, tarsal bones, metatarsal bones, phalanges), and spine. Surgeries were further classified as day surgeries, inpatient elective, and inpatient non-elective surgeries based on the admission status variable in CIHI datasets.

### Statistical analysis

The amount of care provided by orthopaedic surgeons was measured as the number of encounters with orthopaedic surgeons and the number of surgeries carried out by orthopaedic surgeons. The mean number of ambulatory visits per patient was also estimated. Patients with visits for more than one condition in a particular group were counted within each condition, but once when counting the number of patients with visits for the group as a whole. A similar approach was used when counting the overall number of patients with ambulatory visits.

Descriptive analyses were conducted for the number of encounters, number of patients and number of surgeries by service location, condition groups, patient's age and sex. Poisson regression was used to examine the statistical differences in the number of surgeries among condition groups, age groups and sex. A two-tailed p-value of 0.05 was considered significant.

### Ethics

The study was approved by the institutional review board of Sunnybrook Health Sciences Centre.

## Results

The number and characteristics of encounters with orthopaedic surgeons in hospital and ambulatory settings by condition groups is presented in Table 1. There were 1.3 millions encounters with orthopaedic surgeons in Ontario in 2005/06, 86% of which took place in ambulatory settings and the remaining 14% in hospital settings. In both ambulatory and hospital settings the most common conditions seen were injury and related conditions and arthritis and related conditions. Injury and related conditions were seen more often in emergency departments and arthritis and related conditions were most common in patients for day surgeries. The proportion accounted for by arthritis and related conditions in inpatient hospitalizations was similar to the proportion accounted for injury and related conditions.

**Table 1:** Number and characteristics of encounters with orthopaedic surgeons in ambulatory and hospital settings, Ontario 2005/06

	**All conditions**	**Injury and related conditions**		**Arthritis and related conditions**		**Bone and joint conditions**		**Other conditions**	
		
		Number	%	Number	%	Number	%	Number	%
**All settings**	**1,308,109**	**569,375**	**43.5**	**495,552**	**37.9**	**159,555**	**12.2**	**83,627**	**6.4**
**Ambulatory**	**1,125,800**	**496,042**	**44.1**	**420,697**	**37.4**	**141,089**	**12.5**	**67,972**	**6.0**
**Hospital**	**182,309**	**73,333**	**40.2**	**74,855**	**41.1**	**18,466**	**10.1**	**15,655**	**8.6**
Inpatient	87,999	38,710	44.0	36,591	41.6	8,239	9.4	4,459	5.0
Day Surgery	61,831	8,044	13.0	36,806	59.5	8,759	14.2	8,222	13.3
Emergency Department	32,479	26,579	81.8	1,458	4.5	1,468	4.5	2,974	9.2

### Ambulatory care provided by orthopaedic surgeons

Table 2 presents the characteristics of ambulatory encounters by condition and condition groups. The 1.1 million ambulatory encounters with orthopaedic surgeons represent visits made by 438 thousand individuals (35 per 1,000 population), a mean of 2.2 visits per person. Individuals who consulted orthopaedic surgeons for injury and related conditions made on average more visits than any other condition studied. On average individuals with arthritis and related conditions reported 2.1 ambulatory encounters with orthopaedic surgeons with 50% reporting only one ambulatory encounter. Women with arthritis and related conditions and bone and joint conditions had, on average more encounters than men with the same condition, respectively. Women and men had similar number of visits for injury and related conditions or other conditions. In general, the average number of visits per patient increased slightly with age for both men and women and for all conditions studied (Data not shown).

**Table 2:** Ambulatory encounters with orthopaedic surgeons for musculoskeletal conditions in Ontario 2005/06.

**Condition**	Number of persons*	**Persons visiting per 10,000 population**	**Mean number of visits per person**	**Ratio Women: Men**	**Proportion with encounters**		
					**1**	**2 or 3**	**4 or more**
**All conditions**	**438,228**	**350.2**	**2.2**	**1.2**	**47.6**	**36.2**	**16.2**
**Injury and Related**	**211,240**	**168.8**	**2.3**	**1.0**	**38.2**	**43.1**	**18.7**
Fractures & dislocations	116,614	93.2	2.5	1.0	33.6	46.3	20.1
Strains and sprains	96,846	77.4	2.1	1.0	50.3	34.8	14.9
Other injuries	8,982	7.2	2.3	1.0	45.5	35.4	19.1
**Arthritis and Related**	**186,361**	**148.9**	**2.1**	**1.2**	**50.6**	**33.5**	**15.9**
Osteoarthritis	91,505	73.1	2.2	1.5	48.5	33.3	18.2
Rheumatoid arthritis	2,269	1.8	2.3	2.5	49.7	30.6	19.7
Traumatic arthritis	1,502	1.2	2.4	1.0	47.4	31.4	21.2
Joint derangements	54,002	43.2	2.0	0.8	51.4	35.5	13.1
Synovitis	29,761	23.8	2.0	1.2	54.4	32.6	13.0
Ankylosing spondylitis	368	0.3	1.5	1.2	45.3	38.9	15.8
Other arthritis	20,639	16.5	2.2	1.2	52.6	31.6	15.8
**Bone and Joint**	**71,233**	**56.9**	**2.0**	**1.4**	**56.7**	**30.7**	**12.6**
Other spine	27,399	21.9	1.8	1.3	60.3	30.2	9.5
Bone disorders	13,627	10.9	2.0	2.4	58.1	26.8	15.1
Unspecified bone & joint	31,394	25.1	2.1	1.2	52.9	32.9	14.2
**Other conditions**	**20,572**	**16.4**	**2.3**	**0.9**	**51.1**	**32.0**	**16.9**

* Numbers do not add up to totals within each condition group because patients may visit for more than one condition

### Surgery provided by orthopaedic surgeons

Orthopaedic surgeons performed over 140 thousand surgeries in 2005/06 (12 per 1,000 population) (Table 3). Half of them were for arthritis and related conditions, one third were for injury and related conditions and one-tenth were for bone and joint conditions. Three-fifths of all surgeries were inpatient procedures, with only slightly more elective than non-elective surgeries. Day surgeries accounted for two fifths of all surgeries carried out.

**Table 3:** Number of surgeries carried out by orthopaedic surgeons by selected characteristics, Ontario 2005/06.

	**All conditions**		**Arthritis and related conditions**		**Injury and related conditions**		**Bone and joint conditions**		**Other conditions**	
	
	**#**	**(%)**	**#**	**(%)**	**#**	**(%)**	**#**	**(%)**	**#**	**(%)**
**All surgeries**	**144,705**	**(100.0)**	**72,262**	**(49.9)**	**47,449**	**(32.8)**	**15,784**	**(10.9)**	**9,210**	**(6.4)**
Type of surgery*										
Day surgery	59,422	(41.1)	36,284	(50.2)	7,987	(16.8)	8,321	(52.7)	6,830	(74.2)
Inpatient elective	43,340	(30.0)	34,990	(48.4)	-		6,436	(40.8)	1,914	(20.8)
Inpatient non-elective	37,005	(25.6)	806	(1.1)	34,979	(73.7)	864	(5.5)	356	(3.9)
**Surgical groups**										
Joint replacement	36,367	(25.1)	28,278	(39.1)	6,953	(14.7)	1,077	(6.8)	59	(0.7)
Reduction with or without fixation	31,494	(21.8)	370	(0.5)	29,272	(61.7)	1,658	(10.5)	194	(2.1)
Closed repair	24,060	(16.6)	21,804	(30.2)	1,445	(3.0)	800	(5.1)	11	(0.1)
Open repair	16,914	(11.7)	6,945	(9.6)	3,688	(7.8)	5,794	(36.7)	487	(5.3)
Spinal surgery	5,090	(3.5)	1,239	(1.7)	1,198	(2.5)	2,183	(13.8)	470	(5.1)
Other	30,780	(21.3)	13,626	(18.9)	4,893	(10.3)	4,272	(27.1)	7,989	(86.7)
**Anatomic location**										
Knee	61,256	(42.3)	46,940	(65)	10,158	(21.4)	2,245	(14.2)	1,913	(20.8)
Hip	26,398	(18.2)	10,067	(13.9)	13,998	(29.5)	1,582	(10.1)	751	(8.1)
Shoulder/elbow	24,535	(17.0)	9,589	(13.3)	11,740	(24.8)	1,963	(12.4)	1,243	(13.5)
Ankle/foot	18,664	(12.9)	2,387	(3.3)	7,083	(14.9)	7,025	(44.5)	2,169	(23.6)
Hand/wrist	8,762	(6.1)	2,040	(2.8)	3,272	(6.9)	786	(5.0)	2,664	(28.9)
Spine	5,090	(3.5)	1,239	(1.7)	1,198	(2.5)	2,183	(13.8)	470	(5.1)

* Numbers do not add up to total due to missing values

The number of surgeries according to surgical groups is also presented in Table 3. A quarter of those surgeries were joint replacement and over a fifth were reductions with or without fixations, Spinal surgery accounted for a small (3.5%) proportion of all surgeries carried out by orthopaedic surgeons. The most common types of surgical interventions performed in patients with arthritis and related diagnosis were joint replacement and closed repairs; whereas reductions and joint replacement were the most common surgeries carried out on patients with injury and related conditions. Among patients with bone and joint conditions, open repairs was the most common type of surgery performed.

In terms of anatomic location, surgeries of the knee were most common, accounting for over 40% of all surgeries, followed by the hip (18%), the shoulder and elbow (17%) and foot and ankle (12%)(Table 3). The knee was the most common joint operated for patients with arthritis and related conditions and the ankle and foot among those with surgeries for bone and joint conditions.

**Figure 1:**
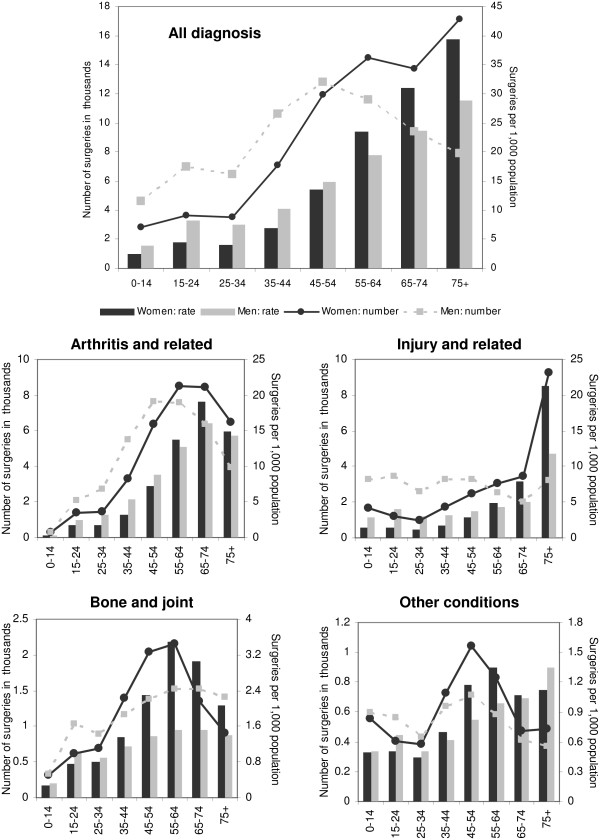
**Number and rate per 1,000 population of surgeries carried out by orthopaedic surgeons for all conditions, arthritis and related conditions, injury and related conditions, bone and joint conditions and other conditions by age and sex, Ontario 2005/06**.

Results from Poisson regression modeling showed that the number of surgeries performed by orthopaedic surgeons varied according to patient's age and sex (p = 0.0088) (Figure 1). The total number of surgeries increased with age among women; however, among men, the number of surgeries increased with age up to the age of 54 years and then showed a considerable decline. Among individuals 44 years or younger the number of surgeries was higher in men than women and among individuals 55 years or older the opposite was observed. The pattern of surgery with age varied according to underlying condition (p < 0.0001). The volume of surgeries for arthritis and related conditions increased with age for men and women until age 64 years and then declined for those 65 years or older The volume of surgeries for injury and related conditions was higher in younger men (less than age 55) and in older women (64 years or older, and particularly those 75 years or older).

## Discussion

Our findings point to the wide scope of the work of orthopaedic surgeons in dealing with musculoskeletal conditions in the population including arthritis, injury and bone and joint conditions in both ambulatory and hospital settings. It begins to set work in the operating room in the context of all patients seen, and points to the major contribution made by orthopaedic surgeons to the ambulatory care of individuals with musculoskeletal conditions.

Overall, the results reported here demonstrate that the number of encounters with patients and the number of patients seen in ambulatory settings is much higher than the number of surgical procedures carried out. Although we were not able to take into account the fact that encounters involving a surgical procedure are likely to be more time consuming than an ambulatory visit, these findings are consistent with the results of surveys of Ontario orthopaedic surgeons indicating that on average they only spend a third of their time in the operating room [16,17]. It is clear from the spectrum of patients seen in ambulatory care by orthopaedic surgeons that patients are referred for advice on non-surgical as well surgical management of a wide range of musculoskeletal problems. It is not known what proportion of patients seen by surgeons eventually have surgery. Analysis of linked data for a number of years would be necessary to establish this. However, the situation reported in this paper is similar to the one in other western nations [12,23-25], and is in line with reports from a range of settings and patients populations that show that 30% or less of patients who see an orthopaedic surgeon are candidates for surgery [26,27]. A potential concern is the extent to which the high proportion of non-surgical patients might lengthen waiting times for consultation with orthopaedic surgeons, and delay treatment of patients in urgent need for surgery. Further studies are needed to address this.

Orthopaedic surgeons have high volume of encounters for injury and related conditions both in ambulatory care and hospital settings, where these conditions account for the majority of encounters. A similar pattern has been reported in the United States where injuries comprised about 40% of all office visits to orthopaedic surgeons [28]. While most of the hospital encounters related either to same day or inpatient surgery, a substantial minority were in the emergency room. The injury and related conditions seen ranged from simple strains and sprains, which were unlikely to need surgical intervention, to fractures and more serious conditions.

Orthopaedic surgeons also see a substantial proportion of individuals with arthritis and related conditions. To set this in overall context, a US study showed that almost a fifth of all physician visits for arthritis were to orthopaedic surgeons [12]: the equivalent proportion in Canada was 15% [5]. In our study, a substantial minority of the patients seen by orthopaedic surgeons had synovitis and unspecified arthritis, conditions which are unlikely to be associated with surgical intervention. Almost half (90 thousand) had a diagnosis of osteoarthritis which far exceeds the total number of joint replacement surgeries (28 thousand). A recent study [29] of patients referred for surgery and assessed by a physiotherapist and an orthopaedic surgeon found that while a third did not require surgery, all patients required appropriate conservative management. Other studies have pointed to deficiencies in the primary care management including both under and over use of non-steroidal anti-inflammatory medications and lack of referral for physical therapy [30].

The pattern of utilization of orthopaedic surgery particularly in the older age groups found here are similar to those reported elsewhere [18,31,32]. The overall surgical rate increases with age, particular for surgery for arthritis and related conditions (including joint replacement surgery) and for injury, particularly fracture. These trends in age specific rates of surgical procedures are important when planning the orthopaedic resources likely to be required to accommodate the needs of the aging population.

In view of concern about future availability of the orthopaedic surgical workforce [9,15,33,34] several strategies could be implemented to increase capacity for orthopaedic surgery. Increasing the number of orthopaedic surgeons is a long-term solution that will not have an immediate effect during the next decades. A strategy for increasing availability of orthopaedic surgeons for surgery would be to ensure that patients who are referred are those who are most likely to need surgery. Enhancement of the capacity of primary care physicians to diagnose and treat musculoskeletal conditions, including musculoskeletal trauma and back and other soft-tissue disorders, is clearly important. In the short term, taking into account the high volume of ambulatory care, the scope of orthopaedic practice could be redesigned to allow individuals not immediately needing surgical services to access to the appropriate musculoskeletal care. For example, it may be possible to increase capacity for orthopaedic surgery by decreasing the amount of clinic time of orthopaedic surgeons by delegating some of the triage and routine follow-up of patients to another musculoskeletal health professional.

Models of care provision involving non-surgical specialists or allied healthcare professionals collaborating with orthopaedic surgeon to provide care to individuals with musculoskeletal problems are being gradually more recognized [34-37]. Studies have shown that specially trained physiotherapists can assess and manage some patients with musculoskeletal conditions while working with orthopaedic surgeons [35,38,39], and data from the UK shows that pre-screening of patients by such therapists can more than double the proportion of patients who need surgery on assessment by the surgeon [26]. The use of physical therapists in these roles also has the potential to facilitate conservative management of patients not yet requiring surgery [40].

A major strength of our study is its high coverage of the Ontario population. Further, we were able to examine data on visits to orthopaedic surgeons in ambulatory settings as well as hospital settings and we considered an extensive list of conditions and surgical interventions. Limitations of the analyses presented here are those inherent in the use of health service data. In particular the surgical data were collected using CCI which is a relatively new classification system. The fiscal year 2005/06 was the fourth year of implementation and coding instructions were still in the process of development[21]. As there were no published data on groupings of orthopaedic surgical codes, one of the key challenges we had was to identify reasonable groups of orthopaedic surgical procedure codes that correspond to what clinicians usually consider orthopaedic surgery.

## Conclusion

This study shows that the role that orthopaedic surgeons play in the management of injuries, arthritis, and bone and joint conditions is wider than surgery. The large volume of ambulatory care points to the major contribution they make to the medical management of these conditions. The number of individuals affected by these conditions is expected to increase as the baby boom generation ages, putting additional pressure in the orthopaedic workforce. Increasing the number of practicing orthopaedic surgeons is a long term solution; in the meantime surgeons and policy makers need to be creative in developing strategies to efficiently accommodate the growing workload without sacrificing quality of care of patients with musculoskeletal conditions.

## Competing interests

The authors declare that they have no competing interests.

## Authors' contributions

MC was responsible for drafting the manuscript and had access to all the data in the study. All authors contributed to the analyses and approved the final manuscript.

## Pre-publication history

The pre-publication history for this paper can be accessed here:



## Supplementary Material

Additional file 1 **Appendix.** Diagnostic codes for condition groups in ambulatory and hospital care databases. Click here for file
