# Supplementary material for: A population-based study of ambulatory and surgical services provided by orthopaedic surgeons for musculoskeletal conditions
Source: BMC Health Serv Res. 2009 Mar 31;9:56. doi: 10.1186/1472-6963-9-56 (PMC2682488; doi:10.1186/1472-6963-9-56)
Supplement: Additional file 1 — Appendix. Diagnostic codes for condition groups in ambulatory and hospital care databases. [file 1472-6963-9-56-S1.doc]

**Appendix**

Diagnostic codes for condition groups in ambulatory care database

| **Condition groups** | **OHIP Diagnostic Code(s)*** |
| --- | --- |
| **Arthritis and related conditions** |  |
| 1. Osteoarthritis | 715 |
| 1. Rheumatoid Arthritis | 714 |
| 1. Traumatic arthritis | 711, 716 |
| 1. Joint derangement | 718, 728 |
| 1. Synovitis | 727-729 |
| 1. Ankylosing spondylitis | 720 |
| 1. Other arthritis | 274, 446, 710, 739 |
| **Bone and joint conditions** |  |
| 1. Other Spine | 722, 724, 737 |
| 1. Bone (including conditions on the foot) | 10, 11, 15, 730-735, |
| 1. Unspecified joint disorders | 781 |
| **Injury and related conditions** |  |
| 1. Fractures and dislocations | 805-821; 831-839 |
| 1. Strains and sprains | 840-848 |
| 1. Other trauma | 824, 829, 850, 854, 869, 879 |

* ICD-9 based and three-digits truncated

Diagnostic codes for condition groups in hospital care databases

| **Condition groups** | **ICD-10 Diagnostic**  **Code(s)** |
| --- | --- |
| **Arthritis and related conditions:**  (Osteoarthritis, Rheumatoid Arthritis  Traumatic arthritis, Joint derangement, Synovitis, Ankylosing spondylitis) | M00- M03, M05–M19, M22-M24, M30-M36, M45-M47,M48.0- M48.2, M48.8, M48.9, M65-M68, M70, M71, M75-M77, M79, M95, M96, M99 |
| **Bone and joint conditions:**  (Other conditions of the spine, bone conditions including foot, an unspecified disorders such as leg cramps) | M20, M21, M25,  M40- M43, M48.3-M48.5,M49, M50-M54, M60-M63, M72, M73, M80-M94, L60.0, L60.2, L84 |
| **Injury and related conditions:**  (sprains and strains, fractures and dislocations and other trauma) | S08, S10-S19, S30, S32-S35, S37-S40, S42-S50, S52-S60, S62-S70, S72-S80, S82-S90, S92-S99,T00, T02-T14 |
